# The Knowledge and Attitude of Diabetic Patients Regarding Oral and Dental Disorders in Kerman Diabetes Clinics

**DOI:** 10.30476/DENTJODS.2020.77878.0

**Published:** 2020-09

**Authors:** Shahla Kakooei, Salimeh Afzali, Masoud Parirokh, Sina Kakooei, Mahshid Mostafavi, Amir Nekouei

**Affiliations:** 1 Neuroscience Research Center, Dept. Oral Medicine, Kerman University of Medical Sciences, Kerman, Iran; 2 Oral and Dental Diseases Research Center, Kerman University of Medical Sciences, Kerman, Iran; 3 Endodontology Research Center, Kerman University of Medical Sciences, Kerman, Iran; 4 Leishmaniasis Research Center, Kerman University of Medical Sciences, Kerman, Iran

**Keywords:** Oral disease, Diabetes, Knowledge, Attitude

## Abstract

**Statement of the Problem:**

Diabetes mellitus is one of the most common endocrine disorders. This disease has devastating effects on many organs and tissues of the body including oral and dental tissues.

**Purpose:**

The aim of this study was to evaluate the knowledge and attitude of diabetic patients about dental and oral diseases.

**Materials and Method:**

In this cross-sectional study, 433 diabetic patients who referred to Kerman Diabetes Clinics were included. Data were collected using a questionnaire consisted of three parts of demographic characteristics, knowledge of oral and systemic complications of diabetes mellitus, and patients' attitude regarding their oral health. Data were analyzed using SPSS version 21 and employing t-test and multiple linear regression analysis. Statistically significant values were considered at *p*≤ 0.05.

**Results:**

The mean scores for the knowledge of systemic and oral complications were 0.80±0.21 and 0.39±0.23, respectively. The mean total knowledge of diabetic patients was 0.53±0.18, and the mean score for the patients' attitude was 0.63±0.11. It was revealed that people with a family history of diabetes did not have significantly greater overall knowledge (*p*= 0.082). Also, people with longer disease duration (*p*= 0.004) and female patients (*p*= 0.05) had significantly a better knowledge and attitude in terms of oral health.

**Conclusion:**

The knowledge and attitude of patients regarding their oral and dental health and diseases were at moderate level, which should be promoted by constant planning and education according to the current needs of society.

## Introduction

With increasing age of patients referring to dental clinics, dentists may visit more patients who suffer from some systemic diseases. In a study conducted in three cities of Iran, about 50% of the patients had at least one systemic disease [ 1 ]. Diabetes mellitus, sometimes known as “silent epidemic”, is one of the important chronic metabolic diseases and a major health problem worldwide. The prevalence of this disease is increasing especially in developing countries [ 2 ]. Currently, diabetes is a major health problem in all developed and developing countries, and its prevalence is increasing day by day in various countries, including Iran. According to the World Health Organization (WHO) report, the prevalence of type 2 diabetes in Iran was 5.7% in 2000 and it will be 6.8% in 2025. Accordingly, the diabetic population in these years will be 1,977,000 and 5,125,000, respectively [ 3 ]. Diabetes type 2 imposes a heavy and increasing burden on medical care all across the world [ 3 ]. The prevalence of diabetes increased from 30 million in 1985 to 135 million in 1995, and it is estimated to be 366 million cases in 2030 [ 4 ]. In addition, many of the new cases of diabetes are observed in developing countries, and it seems that the Middle East will face the highest raise in the prevalence of diabetes in 2030. Furthermore, it is estimated that in 2025, more than 75% of all diabetic patients will be in developing countries. In Iran, more than 3,000,000 people have diabetes and according to WHO estimation, it will be nearly 7,000,000 cases in 2030. With a prevalence of more than 7%, Iran is amongst the areas with the highest prevalence of diabetes in the world. Annually, an average number of 500 thousand individuals will be added to diabetic population of Iran [ 5 , 6 ]. 

Diabetes mellitus is considered as a metabolic disease with many complications such as cardiovascular, neurological, kidney, eye, nerve and mouth complications, and one of the major global health problems [ 7 ]. In addition to its effects on other organs, it can also affect oral health [ 8 ]. Oral symptoms of diabetes mellitus include dry mouth, burning mouth, taste disorders, large thyroid gland, periodontal disease, and bacterial and fungal infections [ 9 , 10 ]. Studies have shown that irreversible complications of diabetes are caused by glycation end products that make changes in cholesterol, albumin, collagen, and hemoglobin, providing the grounds for complications in diabetic patients [ 11 ]. Frequent urination with neurological and pathological changes in salivary glands reduces the amount of saliva in these patients. The oral symptoms of diabetes include decreasing saliva pH, dental caries, gingivitis, periodontitis, mouth irritation, dry mouth, changes in the chemical composition of saliva, oral yeast infections, median rhomboid glossitis (MRG), and oral lichen planus (OLP) [ 12 ].

Having knowledge and proper information about the potential of periodontal diseases and xerostomia in diabetes can be important for the prevention of oral problems in these patients. Studies have also shown that sufficient information about proper oral health behaviors is necessary to take care of one's mouth [ 13 ]. Considering the prevalence of diabetes in Iran (7%) and concerning its consequent oral diseases, this study was conducted to assess the knowledge, and attitude of diabetic patients about dental and oral diseases in Kerman, Iran.

## Materials and Method

In this cross-sectional study, diabetic patients who referred to Diabetes Clinics in two hospitals of Kerman (Shahid Bahonar and Afzalipour) were included. The participants were selected using simple random sampling method. The sample size was calculated using G*Power software version 3.0.1. Based on α = 0.05, effect size= 0.50, and power= 80%, 433 participants were selected for the study.

The inclusion criteria were considered as age between 17 and 75 years, HbA1C > 6.5% or fasting plasma glucose (FPG) >126 mg/dl or 2-hour plasma glucose >200 mg/dl, symptoms of hyperglycemia and hyperglycemia with a random plasma glucose >200 mg/dl [ 14 ], and absence of any psychological problems.

The exclusion criteria were considered as age below 15 and over 75 years, HbA1C<6.5 or FPG<126 or 2-hour plasma glucose or random plasma glucose< 200mg/dl or presence of any mental problems.

Data were collected using a three-part questionnaire. The first part consisted of the demographic and individual characteristics such as age, sex, duration of diabetes, type of treatment, the condition of diabetes, family history of the disease, type of diabetes, and the most recent value of HbA1c and FBS. The second part consisted of the questions related to knowledge about systemic diseases and oral complications related to diabetes mellitus. The third part included questions related to the patients' attitude regarding the prevention of oral and dental diseases such as brushing, flossing, and visiting dentists. The responses were based on three options including “Yes”, “No”, and “I do not know”. 

The validity of the questionnaire was approved by ten experts from Kerman Dental School. The questions' intelligibility was discussed as well. Based on the experts' opinion, 19 questions were considered appropriate and very appropriate. The validity and reliability of the questionnaire were measured by content validity index (0.78), and Cronbach’s alpha (0.80) was at acceptable level. The answers of questions were scored from 1 to 0 as (1) for true answers, (-1) for false answers and (0) for “I don’t know “answers. After summing up the scores, the knowledge of participants were scored as “Good” (0.67,1), “Moderate” (-0.67,0.67), and “Poor”(-1,-0.67). 

The informed consents were obtained from all participants. The participants were assured to keep their information confidential and use them only for statistical reasons. In addition, for semi-literate and illiterate patients, the questions were read to them. The study was approved by the Ethics Committee of Kerman University of Medical Sciences (Ethical code: EC/KMRC/92-52). The results were expressed as percentages and 95% confidence intervals. The data were analyzed using t-test and multiple linear regress-ions with significance level of 0.05 by SPSS version 21.

## Results

In this study, 433 questionnaires were randomly distributed among selected diabetic patients, of which 417 questionnaires were returned. The mean age of patients was 52.1±12.1 years, while 66.5% of them were female and 53.8% had uncontrolled diabetes. At least, 85% of the participants had knowledge about the kidney and eye diseases, paresthesia, and delayed wound healing (Table 1). The participants’ knowledge about the oral complications is presented in Table 2. More than 90% of the patients have mentioned dry mouth (xerostomia) as the most frequent complication (Table 2).

**Table 1:** Knowledge questions about systemic complications of diabetes mellitus.

Type of disease	Yes n (%)	No n (%)	I don’t know n (%)
Kidney disease	386(92.5)	25(6)	6(1.4)
Eye disease	397(95.2)	15(3.6)	5(1.2)
Neurological disease	216(51.7)	121(29.1)	80(19.2)
Heart disease	326(78.3)	63(15)	28(6.8)
Mental disease	286(68.8)	77(18.4)	54(13)
Delayed wound healing	356(85.3)	47(11.8)	12(2.9)
Paresthesia	376(9.1)	26(6.3)	15(3.6)

**Table 2:** Knowledge questions about oral complications of diabetes mellitus.

Oral Complications	Yes >n(%)	No n(%)	I don’t know n(%)
Halitosis	172(41.3)	160(38.4	85(20.3)
Tooth discoloration	114(27.3)	184(44.1)	119(28.6)
Tooth sensitivity	135(32.4)	170(40.8)	112(26.8)
Tooth decay	219(52.5)	119(28.5)	79(19)
Tooth mobility	166(39.7)	162(389)	89(21.4)
Periodontitis	134(34.4)	190(54.4)	84(20.2)
Burning mouth sensation	140(3.7)	216(51.7)	61(14.6)
Aphthous ulcer	107(25.5)	251(60.2)	59(14.3)
Xerostomia	376(90.1)	34(8.2)0	7(1.7)
Taste change	228(54.8)	137(32.8)	52(12.4)
Abscess	109(26.2)	240(57.5)	68(16.3)
Candidiasis	110(26.4)	246(59.1)	61(14.5)

Also, 64.1% of the participants (n=267) believed that blood sugar regulation can prevent oral diseases. In addition, 75% (n=313) of the participants believed that regular dental checkups can prevent the progression or incidence of oral diseases. Moreover, 46.1% (n=192) stated that they used a toothbrush or dental floss regularly, which can improve their oral health (Table 3). 

The results of statistical analyses for questions related to dental and oral health attitude are presented in Table 4. About 60.5% of the participants brushed their teeth twice a day. Unfortunately, 66.8% of the participants did not use dental floss. In addition, more than 50% of them did not check up their teeth annually (Table 4).

The mean score for knowledge about systemic complications was 0.80±0.21, and 4.9% of the patients had poor knowledge about systemic complications, while 15.1% had moderate and 82% had good knowledge. The mean score of knowledge of oral complications was 0.39±0.23, and 50% of the patients had poor knowledge about oral complications, while 35.1% had moderate and 14.9% had good knowledge. The mean score of attitude was 0.63±0.11.

**Table 3:** Knowledge questions about oral disease prevention.

Oral disease prevention	Yes n (%)	No n (%)	I don’t know n (%)
Can blood sugar regulation prevent oral diseases?	267(64.1)	25(6)	125(29.9)
Can regular dental checkups prevent the progression or occurrence of oral diseases?	313(75)	18(4.4)	85(20.6)
Does using a toothbrush or dental floss on a regular basis improve oral diseases?	192(46.1)	74(17.7)	151(36.2)

**Table4:** Attitude questions about dental and oral health.

Practice questions	Answers	n(%)
How many times a day do you brush your teeth?	Once	36(8.8)
	Twice	255(60.5)
	Three times	94(22.9)
Do you use dental floss?	No	279(66.8)
	Some times	70(16.8)
	Yes	68(16.3)
If yes how many times?	Daily	69(16.5)
	Weekly	8(19)
	Monthly	40(9.6)
Do you visit your dentist annually?	Yes	162(39)
	No	255(61)
If yes how many times?	One month	1(0.2)
	Three month	3(0.7)
	Six month	21(12.2)
	Annual	105(25.2)

The mean score was higher in female patients, and there were statistically significant differences in the knowledge of systemic complications (*p*= 0.003) and oral complications (*p*= 0.037) between the two genders. There was no significant difference between total score of knowledge and attitude in both genders. The age variable analysis showed that older people had significantly more knowledge about systemic complications. However, there were no significant differences between all of the dependent variables and familial history. Nevertheless, there was a significant relationship between disease duration and knowledge about systemic complications and total score of knowledge and attitude (Table 5). 

**Table 5:** Relationship between knowledge and attitude with demographic variables.

Dependent Variables	Knowledge about systematic complications		Knowledge about oral complications		Total score of knowledge		Total score of attitude		Total score of knowledge & attitude	
Independent variables	*p* Value	β	*p* Value	β	*p* Value	β	*p* Value	β	*p* Value	β
Age	0.015	0.256	0.419	0.097	0.694	-0.108	0.082	-0.07	0.921	0.014
Sex	0.003	7.158	0.037	5.662	0.487	4.737	0.284	21.549	0.052	6.4
Familial history	0.534	-1.570	0.103	4720	0.082	12.05	0.254	-36.579	0.148	4.678
Duration of disease	0.048	0.388	0.355	0.210	0.928	0.049	0.699	0.362	0.004	0.930

## Discussion

Diabetes mellitus, as the most common metabolic disease, has widespread effects on body including mouth and teeth. The high incidence of candidiasis, periodontal disease, and dental decay in people with diabetes is also one of these effects. This is mainly due to the effects of disorder on gingival crevicular fluid and saliva [ 2 , 10 ]. Studies have shown a relationship between increasing salivary immunoglobulin A (IgA) in diabetic patients and denture stomatitis and xerostomia [ 15 - 16 ]. It is reported that in 2000, 2.8% of world people suffered from diabetes, and estimations show that this will rise up to 4.4% in 2030 [ 17 ]. According to the latest statistics, there were nearly 4 million diabetic patients in Iran. According to the international statistics, this number will become three times more every 15 years [ 18 ]. Poor control of blood glucose due to diabetes leads to nephropathy, retinopathy, stroke, and coronary artery diseases. In addition, studies have shown that diabetic patients are 2 to 3 times more prone to periodontal diseases compared to healthy people. Several studies have proven a reciprocal relationship between periodontal disease and diabetes mellitus. Uncontrolled diabetes mellitus can cause periodontitis but alternatively, treatment of periodontitis can improve blood sugar control [ 16 ]. In this study, the mean age of the patients was 52±12 years, which was close to that reported by Yuen et al. [ 19 ] (57.9±12.8 years). In this study, 66.5% of the patients were women, which is similar to the gender distribution in the study of Delvarianzadeh et al. [ 20 ]. The mean duration of disease since diagnosis in patients was 8.8±6.4 years. As age increases, the incidence of complications of the disease also increases [ 21 ]. Another study has reported that the mean duration of diabetes complications is 10.5 years [ 22 ]. Consistent with the results reported by Eldarrate et al. [ 23 ] and Allen et al. [ 24 ], the results of this study showed that diabetic patients' knowledge of other systemic diseases associated with diabetes is more than their knowledge of oral disease. The mean score for knowledge of systemic and oral complications was 80.4± 21.4% and 39±23.3%, respectively. The percentage of participants who were aware of increased risk of eye, kidney, and heart diseases was much more compared to the knowledge of gum disease, tooth decay, and fungal infections.

According to the results, the diabetic patients' knowledge about the possibility of developing oral diseases such as gum disease, tooth decay, and fungal infection caused by dry mouth was very inadequate. The results of this study showed that 50% of the participants had a low knowledge about the oral complications of diabetes. Several studies have reported that diabetic patients' knowledge about oral health and oral complications of diabetes is inadequate [ 25 , 27 ]. In this study, 90.1% of the participants were aware of the relationship between diabetes and dry mouth. Kakoei et al. [ 28 ] showed that xerostomia had the most important factor on oral health impact profile (OHIP) in diabetic patients. In the of Eldarrate et al. [ 23 ], it was shown that more than 70% of the participants suffered from dry mouth while they were not aware of the harmful effect of dry mouth on health. 

Yuen et al. [ 19 ] showed that only 30% of diabetic patients were aware of the effect of dry mouth on health. Only 64% of the participants in this study believed that the blood sugar regulation could prevent oral diseases. Insufficient knowledge of the relationship between glycemic control in patients with diabetes and periodontal disease was remarkable in this study. It is clear that studies on the knowledge of the relationship between diabetes and periodontal disease in diabetic patients are not sufficient. It seems that the knowledge of such relationships should be strengthened.

In this study, only 46% of the patients believed that brushing or flossing could improve the disease treatment process. In addition, it was revealed that follow-up treatments of periodontal diseases significantly improved blood sugar control in patients with type 2 diabetes [ 29 ].

In the present study, 52.9 % of the participants had an average attitude level in preventing oral and dental diseases. In addition, 60% of the participants brushed their teeth only once a day, 22% brushed their teeth twice a day and 8% did not brush their teeth at all. In addition, more than 66% of the participants did not floss their teeth. Previous studies have shown that only 22.9% of the participants brushed their teeth twice a day, and the majority (73.6%) did not floss their teeth [ 17 , 29 ]. Given the important relationship between periodontal disease and diabetes, effective behaviors for preventing periodontal disease such as brushing, flossing, and regular dental visits not only can have positive impacts on the treatment of periodontal diseases, but they should be performed completely in order to maintain the health of diabetic patients [ 17 ].

The results of data analysis showed that 39% of the female patients visited a dentist for check-up, which is consistent with the results of previous studies [ 23 , 24 , 30 ]. It was also revealed that 85% of the patients had not received any advice from a specific source to follow routine dental checkups. Physicians (7.4%) and friends (2.9%) were the main sources of suggestions for visiting a dentist. A study by Al Habashneh et al. [ 31 ] showed that for 50% of the patients, TV and internet were the main cause of referral to dentists. In another study, only 5.2% of the patients were advised by their physicians to visit a dentist [ 17 ].

Studies have shown that patients, who visited their dentists regularly, had a better understanding of the relationship between oral diseases and diabetes [ 17 ]. It is obvious that more knowledge of oral complications of diabetes can help patients have a better attitude with respect to prevention and treatment of oral diseases. For this reason, public health education aims to improve patients' attitude via increasing their knowledge, and to help them take care of themselves. In recent studies conducted in Iran, it has been reported that the knowledge and attitude of diabetic patients about diabetes oral complications were poor, and none of the methods used in teaching patients about this problem was successful, hence, it seems that further studies should be done on this issue [ 31 , 32 ].

One of the potential reasons for the inadequate patients' knowledge may be the lack of awareness of the relationship between diabetes and periodontal disease among physicians and dentists. Hence, it is better to evaluate the awareness of health personnel regarding the relationship between diabetes and oral health in future studies. In addition, the information of medical and health personnel should be regularly updated [ 17 ].

## Conclusion

According to the results, diabetic patients' knowledge and attitude of their oral and dental health is at moderate level. The analysis of the responses of the participants showed that female patients and those with longer diseases duration have a better knowledge and attitude of oral and dental health. Therefore, providing knowledge of periodontal and oral diseases to the public, especially diabetic patients can be effective in improving the attitude in this area. 
